# Silo Effect a Prominence Factor to Decrease Efficiency of Pharmaceutical Industry 

**Published:** 2013

**Authors:** Hossein Vatanpour, Atoosa Khorramnia, Naghmeh Forutan

**Affiliations:** a *Department of Pharmacoeconomy and Pharmacy Management, School of Pharmacy, Shahid Beheshti University of Medical Sciences, Tehran-Iran.*; b *Pharmaceutical Sciences Research Center, Shahid Beheshti University of Medical Sciences, Tehran, Iran.*; c *Students Research Committee, Shahid Beheshti University of Medical Sciences, Tehran, Iran. *

**Keywords:** Silo, Fragmented, Pharmaceutical, Integration, Supply, Management

## Abstract

To be sure, all the industries try to be involved in globalization with a constant trend to find out ways to increase productivity across different functions within an organization to maintain competitive advantage world. Pharmaceutical industries are not exceptional and further are based on fragmentation. So these kind of companies need to cope with several barriers such as silo mentality that may affect efficiency of their business activity. Due to eliminate a part of resources such as raw materials, new molecule developed, financial and human resources and so on, companies can gradually loss their competitive potentials in the market and increase their expenses.

Furthermore, to avoid any business disturbances in financially connected companies due to silo effect, they should arrange their management to integrated organization form. Otherwise, actions taken by one business member of the chain can influence the profitability of all the other members in the chain. That is why recently supply chain has generated much interest in many business units.

In this paper, it has been tried to investigate the different aspects of silo effect which can affect integrate supply chain.

Finally, a fluent communication, high level of information exchange, fragmentation management, cross-functional control in a supply chain management format are needed to reduce or control silo effect within entire chain of the holding company by Supply chain management.

## Introduction

In the world of product development, and specially management requirement, one may hear a lot about building a “central repository” or a “single system of record”. But, why does that matter? What’s the real value in creating a central hub of product intelligence? It all comes down to productivity. Without a solution that keeps everyone and everything connected, organization is vulnerable to the common issues that plague product development teams. The top three productivity killers: Too much time and money wasted searching for the latest information. Information silo kill productivity and clog the arteries of innovation. Social capital is lost with employee transitions.

How much more productive could your team be if you had all of your product intelligence ideas, feature requests, requirements, design specifications, analysis documents, release plans, defects, trace relationships plus all the related conversations that happen during the development process in one system that’s accessible, organized, and connected at all times?

The ultimate goal in supply chain management (SCM) is to create value for the end customers as well as the firms in the supply chain network. To accomplish this, firms in the supply chain network must integrate process activities internally and with other firms in the network. The term process integration means coordinating and sharing information and resources to jointly manage a process. Integration is process of redefining and connecting parts of a whole in order to form a new one (1).

Process integration can sometimes be an extremely difficult task, because it requires proper training and preparedness; willing and competent trading partners; and, potentially, a change in one or more organizational cultures. However, the benefits of collaboration and information sharing can be significant: reduced supply chain costs, greater flexibility to respond to market changes, less supply chain safety stock, higher quality levels, reduced time to market, and a better utilization of resources (2). Every organizational model either medium or large sized, divides its employees into business units or departments and pharmaceutical not exception. Each fragmentation consists of people and cross-functional^1^ team that they create to own area. The ultimate success of the business requires cross-functional integration and management’s abilities within the firm and across the network of firm to integrate the company’s intricate network of supply chain.

Our present environment and the opportunities for innovation requires more integrated thinking and cross-functional solutions, but many efforts like silo effects still occur independently, isolated from other’s insights and perspectives. The sharing success and working for the greater good are meaningful and appealing to organization, but individuals frequently act out of self-interest.

If everyone does his or her job well, everyone succeeds and in fact, if there is a problem anywhere in the organization, everyone fails. Everyone is responsible for each other›s work in any organization, it›s not only important that everyone do what they are supposed to do, everyone also needs to work together.

By understanding the supply chain management processes and how they should be implemented, executives will be able to create more integrated supply chains which will lead to higher revenues and increased profitability for all member firms.

Since 2010 the phrase “silo effect”, has become popular in the business and organizational communities, it refers to a lack of communication and common goals between departments in an organization. It is of course opposite of system thinking in an organization (3). Critics of silo contend that managers serve as information gatekeepers, making timely coordination and communication among departments difficult to achieve, and seamless interoperability with external parties impractical.”

*Fragmentation and fragmented industry*

An industry in which no single handed enterprise has large enough share of the market to be able to influence the industry›s direction and the other way an industry in which there is no clear leader in market share, and no company determines the direction in which the industry is going. It is more difficult to predict market trends for fragmented industries than for industries with a clear market leader.

*Fragmentation in pharmaceutical industry*

The global pharmaceutical industry consists of thousands of companies, including biotech firms, generic drug-makers, contract research organizations, wholesalers and retailers. The pharmaceutical industry is relatively fragmented, with the biggest company, Pfizer, holding less than 10% of the global market.

The pharmaceutical industry is awash with sales and marketing data, but getting instant access to this information to solve business problems is often fraught with difficulty. An immediate and fundamental obstacle is that answering even simple business questions usually requires several sources of disparate data to integrate.

Data providers themselves do not have a completely integrated view of the sales and marketing pharmaceutical universe; some specialize in primary care markets, some in secondary care and others have limited coverage in both. In addition, Data providers have historically developed their products and solutions around single data sources, focusing on one particular outlet or market sector at a time.

Furthermore, this approach is often inherent in the way the data provider’s business is organized in contrast to the consumer’s need for a broader view of the pharmaceutical universe. Internally, pharmaceutical companies also have operational database systems focused on single data sources, such as ETMS, CRM, financial or HR data – all of which can be applicable to the sales and marketing teams. These factors combine to hinder the provision of a full picture of all the sales and marketing data in one easily accessible database. Another challenge is that historically the technology available within open-market reporting and analysis tools has not been able to cope effectively with the complex pharmaceutical data models. This has led to data providers developing various proprietary tools that handle only their own particular types of data.

Finally, pharmaceutical companies have struggled to bring real pharmaceutical data and technical knowledge together internally to implement a truly integrated picture of all their possible sales and marketing data sources. Unlike other industries such as finance and manufacturing, little outside specialist knowledge has been available to help. Hence, pharmaceutical companies are reliant on their own internal IT and marketing teams for their knowledge of the business problems, further fragmentation in data sources occurs between national and sub-national data, underlying data and appropriate technology.

The pharmaceutical industry faces a common problem in the fragmentation of sales and marketing data. This has led to: Significant amounts of time being spent inappropriately preparing and formatting data. Proprietary solutions handling only one data source at a time. Opportunities lost because the behavior and characteristics of pharmaceutical markets cannot be analyzed quickly enough to ensure that sales and marketing investments can be channeled efficiently.

*Silo effect, the main issue in fragmented industry*

The term “Silo” is quite commonly used in literature on organizational performance to describe inwardly focused organizational units where external relationships are given insufficient attention. Breakdowns in communication, co-operation between unit participants and other stakeholders, and the development of fragmented behavior, are common features. The result is that the organization falls short of making its best contribution to the needs of immediate and wider groups with an interest in the unit’s continued good performance (4).

Silo can arise within organizations. In a paper quoting a 2003 Survey on Leadership Challenges by the American Management Association. Florence Stone notes that:

“….. *Getting people who have different agendas to work together is amongst the biggest obstacles facing business today” *(5).

Silo can also arise between organizations. In a recent paper Conrad Guelke notes that:

“…. Organizational parochialism is characterized by a lack of co-operation between [and within] agencies. In a corporate environment where decision-making is being increasingly “unbundled”, and business unit fiscal performance is the priority, the values of teamwork and co-operation are often neglected” (6).

Silo may be defined as groups of employees that tend to work as autonomous units within an organization.

On a farm, silo prevents different grains from mixing. In an organization, the silo effect limits the interactions between members of different branches of company, thus leading to reduced productivity.

The silo effect is a phrase that is popular in the business and organizational communities to describe a lack of communication and common goals between departments in an organization (7). Silo maybe defined as groups of employees that tend to work as autonomous units within an organization. They show a reluctance to integrate their efforts with employees in other functions of the organization.

*Silo can distract disciplines of organization*

Although not a major focus in this report, silo behavior can also arise between disciplines and occupations.

In an article on management cultures and learning, Edgar H. Schein notes That “occupational communities” (fishermen and miners are quoted) often develop similar attitudes within their occupations irrespective of national origin (8).

The smooth running of complex organizations such as hospitals and Universities, for example, demands very high levels of understanding and co-operation between professional groups with different expectations and training. The tensions that can arise between these groups can reduce organizational performance unless well-managed.

Silo, whether within or between organizations or between disciplines, have their origin in human behavior. The term “silo mentality” is often used describe individual and group mindsets which can be divisive within and between organizations and which are most often manifested as communication barriers creating disjointed, disconnected and detrimental way of working. It is helpful to think of silo existing in degrees along a continuum.

*Silo mentality/Silo effect*

A mind-set present in some companies when certain departments or sectors do not wish to share information with others in the same company. This type of mentality will reduce the efficiency of the overall operation, reduce morale, and may contribute to the demise of a productive company culture. Nowadays when somebody does something better or faster, this may waste, due to the respecting the silo mentality, the second phase of the process is made so it can start at an established moment with an established outcome coming from the previous, so… the positive effect is lost (9). When group of firms are working together a delay in any steps it will transfer to entire chain, then according to Wisner et al. “I win, you lose” silo mentality manifests itself in the form of using cheaper suppliers, paying little attention to the needs of customers and assigning few resources to new products and service design. Eventually, these firms will create quality, cost, delivery timing, and other customer service problems that are detrimental to the supply chain (10). Describes silo mentality as the most significant obstacle to overcome in supply chain management of most companies. Internally, the silo effect can also be present among department (11).

Too often, companies do not consider the impact of their actions on their supply chains, long term competitiveness and profitability. An “I win, you lose”, silo mentality can stand out when using the cheapest (or hungriest) suppliers, paying little attention to the need of customers, and assigning a few resources to new product and service design. Particularly with companies involved in global supply chains, silo mentalities can crop up, stemming from cultural differences (12).

Conditions that may be conducive to the establishment of silo mentality are covered in a 2004 paper by Florence S. (13). Summarizing a survey on internal collaboration by the American Management Association, notes that these conditions may include: The attitude of unit managers. Indifference to other departments’ needs. Strong departmental priorities relative to corporate priorities. Poor project management. Geographic separation. Isolation mind-set. Financial rewards based on individual unit results. Policies/ procedures that make co-operation difficult.

*Information Silo*

An information silo is a management system incapable of reciprocal operation with other related management systems. The expression is typically applied to management systems where the focus is inward and information communication is vertical.

In pharmaceutical business information, analysts have to contend with delivered through a numbers of different proprietary canal with different data provider. Indeed, some providers supply one tool to deal with one type of data and another tool to deal with a different type of data. Moreover, pharmaceutical companies have their own internal tools to extract the data from a variety of different sources.

Each new report or analytical request from management may mean hours, or even days, of collating data to answer what appear to be simple business questions.

Even for the companies who have invested significant amounts of time and money on building a data warehouse, it is often the case that adding new, or modifying existing, data sources can lead to significant expense and change that does not fit within the timescales of the business.

Factors such as bridging and complex data modeling all contribute to the time it takes to integrate the disparate data sources into one database in pharmaceutical industry, business consumers of the data such as medical doctors, senior managers, brand managers, sales manager, product manager, distributor managers and sales representatives simply cannot afford wait.

The prominent problem that pharmaceutical problem face is a disproportionate amount of a business information analyst’s time is spent integrating data into reports using tools and technology not really suited for the job. This leaves the analyst with little time for real analysis of cause and effect in markets- relegating important analysis of the behaviors of a market to a “nice to have”. Thus pharmaceutical faces uninvited to information silo effect.

*Silo effect classification*

Silo effect can classify according to the spreading direction within organization or chain. In doing so, silo has barriers fragment organizations. These barriers create an ‘us and them’ mentality which makes boundary crossing difficult, and often causes major anxiety in employees having to attend meetings with or visiting other departments, sites or teams.

*Information Silo*

An organization face obstacle for flow information entire chain and these barriers can happen to managers with employees or within employees together, that categorize it to: Vertical Silo; Horizontal Silo; Silo Mentality/Silo Thinking.

Silos are not really physically present in organization; they exist in mind of employees who have a shared impression of its reality (Diamond and Allcorn, 2004, 2009). They believe if they keep other out, they can make safety and comfort environment for themselves. They make specific barriers within area for themselves and who are ‘not like us’.

John S. Carroll suggests that employees at different levels in a given hierarchy can have different understandings and may not communicate easily (14).

Davise discusses how an organization with silo mentality will “*look to their own …. Interests rather than that of the … (organization) as a whole*” (15).

*Financial Silo*

The chain of firms (supply Chain) that they are working together for a common goal, maybe due to the lack of trust, some firms of this chain save financial of resource for own credit and they ignore the benefit whole chain.

*Supply chain*

The supply chain’s phrase initiated over the two last decades, while all the businesses face severely competition environments. In such competitive environment, any frustrating times will make irretrievable effect on whole entire firms that are linking together and contribute to make beneficial businesses. As mentioned ahead, pharmaceutical industry is a fragmented industry that all parts should work harmoniously together to the entire chain gain maximum benefit. Consequently supply chain management and shareholder values are closely linked, and supply chain manager play a vital role to integrate the chain.

Supply chains need to provide an adequate service level (minimizing stock-out costs) while controlling costs of holding, ordering, transporting, and purchasing.

The continuous relationships make supply chains more efficient and the flow of information will be without obstacle that might include lower purchasing costs for the core supply chain member, which may pass these savings on to customers or better service to customers, thus the vendor (or supplier) has more precise information about customers demand, thus they can have more control on their operations consequently they will have more efficiently chain.

*Supply chain integration*

The purpose of supply chain management is described by Kaufman as follows “… remove communication barriers and eliminate redundancies” through coordinating, monitoring and controlling processes (16). The integration of supply chains has been described by Clancy as:

… Attempting to elevate the linkages within each component of the chain, (to facilitate) better decision making [and] to get all the pieces of the chain to interact in a more efficient way [and thus] … create supply chain visibility [and] identify bottlenecks (17).

The bass of integration can therefore be characterized by cooperation, collaboration, information sharing, trust, partnerships, shared technology, and a fundamental shift away from managing individual functional processes, to managing integrated chains of processes (18).

According to the research, one of the prominent effects that make obstacle for integration within organization is silo effect. Silo effect can appear as silo mentality, financial silo, information silo, vertical silo and horizontal silo in a chain of firms.

*Cause silo effect in fragmented industry*

The silo effect has the propensity to exist throughout a company, or between subsidiaries within a wider corporation, resulting in division and fragmentation of work responsibilities within the organization. Departments and business units can fragment into even smaller silos based on strong personal bonds, and areas of commonality that differentiate groups of employees from the rest of their departments, or lack of communication causes departmental thinking to hide ideas from other departments. Organizational silos don›t just appear spontaneously, they›re created by a mix of mindset, culture, and process factors that many associations share. The silo effect gets its name from the farm storage silo; each silo is designated for one specific grain.

Silos are also an offshoot of decentralized management. Once one sector starts to see its own goals as more important than those of the organization as a whole, and when individualism predominates over team spirit, silos emerge. The followings are some causes that crease silo effect within organization: 

*Different departments, different goals*

In silo formation, it is important to realize that individual divisions often have necessary, but conflicting priorities. Usually, when organizations turn into silo, it›s because goals, and any compensation tied to those goals, reinforces silo-oriented behavior. As mentioned above, pharmaceutical industry is fragmented essence, each of them has their own authority and objective too. The common conflict that is more highlighted in pharmaceutical industry is between pharmaceutical shareholders’ objectives and medical services that can serve to society.

*Promotion and over incentives*

In those chains are often major incentives for an employee to restrict most personal interactions to their own division. Employees generally report to members of their own division. When they find a lot of win/lose trade-offs. For example, if the budgeting process starts with a fixed pie and when one of them gets something it comes out of the other›s budget, it›s win/lose, which reinforces organizational rivalry. Result**: **Silo

In the other hand there is often major incentives for the deportment to restrict most personal interactions to their own area. The departments generally report to members of their own portion. Performance is appraised and compensation determined based mostly on perceptions from within their departments and maybe finished projects is counted as successful when they finish on time no matter what, while Operations is counted as successful based on system stability and performance. This means although the ultimate result has an incentive for entire chain but keeping all new codes out of production as long as possible shouldn’t neglect. Result: Silo.*Different Education levels, different concepts* Significantly, when left to their devises, employees within an in-group form and entrench their own sub-culture that becomes distinct from the rest of the organization (The term “sub-culture refers to the creation of new cultural characteristics within a subgroup of an organization). This sub-culture makes it increasingly difficult for other members of the company to integrate into the group. Outside employees may have difficulty in understanding and articulating this culture and be unable to conform to the social norms it requires.

Employees within a given or in-group often socialize more with each other even at communal office functions. Members of a given out-group maybe and feel unwanted by the in-group within a social context. Employees will tend to feel most comfortable in their own division with their friends. Alternative sub-cultures are hard to crack, different and less inviting. Hence organizational silos are created.

*No direct access end user comments*

In Pharmaceutical industry access to end customer almost not possible, it is a prominent conflict that who is the real end customer in pharmaceutical industry. Is it the medical doctor who prescripts medicine for patients? Or is it the patients visit medical doctors to get prescription for own disease?

*Complicated regulatory and Team cross-function authority*

The pharmaceutical industry develops, produces, and markets drugs or pharmaceuticals licensed for use as medications. Pharmaceutical companies are allowed to deal in generic and/or brand medications and medical devices. They are subject to a variety of laws and regulations regarding the patenting, testing and ensuring safety and efficacy and marketing of drugs. High level fragmented area and serious regulatory test for each portion and strong authority for them, easily push pharmaceutical industry to face silo effect.

*Fast expansion companies*

When a company experience fast growth, it tends to become more segmented, the company’s core values, strategies, mission, and vision will not clearly communicated and understand throughout the growing organization.

*Lack of trust*

In organization are no trusts at all, their employees appear to be out to undermine each other more than they›re out to be successful at their own responsibilities. Worse, the attitude is contagious. The people reporting to them act just like they do. Thus entire chain it will be no trust, no ability to collaborate ... nothing. The supervisors accept a lot of blaming, which seems to have become our core competency.

*Knowledge be superiority*

As pharmaceutical industry is knowledge basis some individuals guard information and knowledge as a way to garner and apply power. They believe as long as we have knowledge for ourselves, we will win and the other loses for this reason they show a reluctance to share information with the others.

*Communication barriers*

Silo formation is undesirable as it creates barriers to communication among divisions. This facilitates divisions working in isolation, which negatively impacts the work process since there is a lack of integration between functions. This is particularly serious in service-based organizations like pharmaceutical industry that compromise on the ability to offer an integrated solution. In virtually every company where silo effects are present, various broad organizational objectives and goals are not optimally achieved; because the collective brain power and work potential of the organization cannot be fully harnessed.

*Minimal Co-operation between areas*

Employees do not co-operate with and assist other departments in the company as much as they should, costing the firm time, effort and money. Importantly within a company or Group that offers a variety of products or services, employees may be reluctant to refer work to other sectors of the company.

*Contagion*

When Silo effect happens in one part of chain unfortunately it will spread within entire supply chain.

*Over care about own department*

When staff members more than proud and care about their functional responsibilities, might it’s great but taken to the extreme it can result in close-mindedness against input and feedback from others outside their department. Thus the chain will interrupt and in result the chain efficiency diminish.

*The Psychology of ‘feeling a victim’*

This is unfortunate because it is difficult for existing members of the firm to break down the communication barriers in light of a pervasive feeling of helplessness. The individual employee’s perception of the problem tends to be that it is insurmountable. Employees each feel that it is not their fault and exists outside their personal control. The communication breakdowns lead to errors and finger pointing, as employees want to distance themselves from errors, and avoid taking responsibility for mistakes that could impede their career progression or credibility within the Company. There is broad recognition of a “communication problem” of which everyone feels a victim.

*Silo affect difficult phenomena to eliminate*

Once silo are constructed, they cannot be easily eliminated. People within silo have a propensity to form lasting friendships. Their subcultures become entrenched. They often socialize after hours with people within their silos and create a social distance between themselves and the rest of the company. As time passes, this distance between silos becomes more difficult to narrow.

*How to recognize Silo Effect?*

The answer to blow question help you that your organization is in silo or not:

Are meetings (a) run top-down; or (b) do they include a free exchange of ideas and information?

Do facilities in multi-chain organization (a) have wildly different corporate cultures; or (b) do they operate in a similar fashion, reflecting the core values of the organization?

Are there (a) communication challenges, such as difficult to reach team members or lack of an effective correspondence system; or (b) is it easy to relay information between facilities, departments and staff members?

Are customer- *i.e*., residents and families- (a) angry, anxious, and demanding; or (b) do they like a valued part of the team?

*Control the Silo effect*

Refuse to allow people to go to their separate corners. Encourage people to meet regularly to share what they are learning. Have the courage to call out when one part of the organization is struggling and find a way to fix it together.

System thinking2 has been defined as an approach to problem solving, by viewing «problems» as parts of an overall system, rather than reacting to specific part, outcomes or events and potentially contributing to further development of unintended consequences.

• Streamline procedures so that data is easily collected once rather than multiple times.

The flow of information play vital role in pharmaceutical, thus those technologies and facilities make the flowing information smoother will be increase efficiency and productivity of organization.

• Share best practices among facilities, departments and units. Human resource training can increase creative and collaborative thinking within supply chain.

• Encourage feedback and involvement in new approaches. Getting feedback help the boss breaks through his silo by stating he is looking for more information, and he can better touch to their employees.

• Have a facility “suggestion box” – and take the suggestions seriously.

A suggestion box allows people to anonymously share their ideas and experiences and can be a valuable source of information. Look beyond the complaints/suggestions for the underlying problems. Organizations can disrupt the «I own this area» mindset noted above by encouraging (or requiring) broader and more diverse input into decisions and program development. In short, allow more people to have voice. Staff specialists don›t relinquish their expertise to the wisdom of crowds; rather, they allow their knowledge to be informed and amplified by others› insights and perspectives.

• Listen to the resident and family councils. In this competitive atmosphere, the more satisfied the residents and families are, the more likely they’ll refer friends and neighbors to you. By raising concerns, they’re doing you the favor of pointing out areas that, when addressed, can become selling points for your facility. Train your team on customer service techniques and watch your organization flourish.

• Get feedback from all members. That allows everybody involved in the implementation benefits.

• Meetings and conversations. An easy way to walk the talk of breaking down silo, is to consider who we currently walk and talk with and change our patterns of interaction, a strategy suggested by Cynthia D›Amour. Meetings create and share meaning, and changing their patterns is a simple way to bridge silo. That being said, meetings are also time drains and can be the bane of a staffer›s existence. To avoid negative repercussions from broadening meeting audiences, be sure to:

Share agendas in advance with timed items.

Allow individuals to attend only the portion of meetings where they can make a contribution.

When appropriate, let people share input in advance without attending.

Ensure meeting facilitators are appropriately skilled to manage conversations, particularly ones involving larger and more diverse groups of participants.

## Conclusion

According to our investigation, pharmaceutical industries are severely fragmented and so silo effect is more highlighted in them rather than other industries. Pharmaceutical industries consist of several authorized units that led to silo effect which causes loss of competition in the market as well as expenses augmentation. On the other hand to keep their position in the market they need frequent innovation and highly equipped management for flow of information that playes a prominent role in pharmaceutical companies successful mission.

Silo effect or silo mentality is a factor that leads to redundancies, poor communication, reduced trust, and often keeps one department or group against another in the company. It is important to understand how silos forms, the impact they have upon workplace culture, morale and productivity, and most importantly, how to move past the silo mentality towards unified, high performance teamwork.

Further silo effect is long last barrier in a part of organization that will spread along whole organization. An organization’s first line of defense against an unhealthy silo mindset is the hiring process that hard attention to this part can be the best practice. But some silo beliefs and mindsets are bound to linger, and organizations should attempt to replace them with alternative thinking whenever possible.

Silo existence isn’t a binary, black and white mater. Most organizations and organizational units will have some silo characteristics at any given time. But silos become problematic when they are develop to a point that good performance suffers. The challenge is to indentify silos that are problematic or that threaten to become so, and to identify and take remedial steps.

Reduction or control of silo effect can fosters innovation and increases productivity by unlocking the information within portions that they need for cooperation and collaboration. Of course its control also gives a good chance to use the resources on time, will reduce the expenses and increases the competition potential of the company. Suggested approach to control the silo effect is supply chain management that will cause a fluent flow of information in all parts of the company.
